# Learn by Doing: A Model for Incorporating High-Impact Experiential Learning Into an Undergraduate Public Health Curriculum

**DOI:** 10.3389/fpubh.2019.00031

**Published:** 2019-02-26

**Authors:** Margaret L. Chorazy, Kimberly S. Klinedinst

**Affiliations:** Undergraduate Programs, College of Public Health, University of Iowa, Iowa City, IA, United States

**Keywords:** public health education, undergraduate education, undergraduate public health, bachelors of public health, experiential learning, applied learning

## Abstract

Many accredited schools and programs of public health integrate experiential learning into the capstone experience for undergraduate public health majors; thus, the experiential learning capstone must be both cumulative and integrative. A goal of experiential learning is to foster the application of concepts and skills learned in the classroom to real-world public health situations. Students may benefit from earlier opportunities to engage in high-impact experiential learning activities. Therefore, the University of Iowa College of Public Health developed an experiential learning requirement that is separate from the capstone course. Our students' experiential learning activities do not need to be cumulative across the entire curriculum, but they should be integrative. Public health undergraduate students at the University of Iowa must successfully complete at least one of the following experiences in public health: research, internship, service learning, or global learning. This article will provide a model for the creation of an experiential learning program for undergraduate public health students that is separate from the culminating, capstone experience.

## Background and Rationale

Public health education at the undergraduate level continues to grow following national recommendations to increase the proportion of 4 year colleges and universities that offer public health degree programs such that all undergraduates have access to public health education (1–3). The 2003 Institute of Medicine report also drew attention to the importance of training for the public health workforce (3).

The Council on Education for Public Health (CEPH) accredits undergraduate public health programs. An emphasis on public health training for the workplace or further education is evident in the current accreditation criteria for undergraduate programs in schools and programs of public health and standalone baccalaureate programs^1^^,^^2^. The criteria require that students attain two foundational competencies including (1) to communicate public health information, in both oral and written forms and through a variety of media, to diverse audiences, and (2) to locate, use, evaluate, and synthesize information. Furthermore, undergraduate public health curricula and overall undergraduate curricula should expose students to cross-cutting concepts and experiences that will contribute to success in the workplace, post-baccalaureate education, and life-long learning. Finally, students must have opportunities to integrate, synthesize, and apply knowledge through cumulative and experiential activities.

High-impact educational practices, including experiential learning activities, have been incorporated into the critical component elements identified by the Association of Schools and Programs of Public Health^3^ and in the Liberal Education and America's Promise (LEAP)^4^ learning objectives promoted by the Association of American Colleges & Universities. However, few published articles provide a model for experiential learning activities in public health education, and many of these focus on graduate education in public health (4–10).

Many accredited schools and programs in public health integrate experiential learning into the capstone experience for undergraduate public health majors; thus, the experiential learning capstone must be both cumulative and integrative. A goal of experiential learning is to foster the application of concepts and skills learned in the classroom to real-world public health situations. Students may benefit from earlier opportunities to engage in high-impact experiential learning activities. Especially as undergraduate education is a time of exploration, encouraging experiential activities earlier in the academic career can help students to figure out what they want to do or do not want to do after graduation. Therefore, the University of Iowa College of Public Health developed an experiential learning requirement that is separate from the capstone course. Here, we provide a model for the creation of an experiential learning program for undergraduate public health students.

## Pedagogical Framework and Principles

The origins and development of experiential learning as a model for student learning can be found in the works of Dewey and Kolb among others (11, 12). As opposed to learning that occurs in the traditional classroom, experiential learning relies on experience as the source material and thoughtful reflection to facilitate learning. Experiential education is the philosophy which guides experiential learning. According to the Association for Experiential Education (AEE), experiential learning “occurs when carefully chosen experiences are supported by reflection, critical analysis, and synthesis^5^”. The AAE identifies 10 additional principles of experiential education practice which highlight learner independence, active learning as a process, intellectual and social engagement, and the personal nature of growth through experiential learning. The National Society for Experiential Education (NSEE) has also identified eight principles of good practice for experiential learning activities^6^. We utilized the principles of the AEE and NSEE as we developed the academic framework of our experiential learning program to support guided reflection by public health students.

## Learning Environment

### Setting

The University of Iowa is a public research university, a member of the Association of American Universities and the Big Ten Conference, and home to 33,564 students. The university's academic mission is carried out by 12 colleges, which offer undergraduate, graduate, and professional training. Three-quarters of the students enrolled at the University of Iowa are undergraduates. The University of Iowa's Strategic Plan for 2016 to 2021^7^ includes a goal to “provide a transformative educational experience that educates all University of Iowa students to be engaged citizens” and a strategy to achieve this goal by preparing students to be “experts in their disciplines and leaders in their field” through investment in high-impact practices that “promote critical thinking, problem solving, discipline-based knowledge, analysis, creativity, synthesis, and perspective taking.”

The College of Public Health at the University of Iowa has a long history of outstanding graduate education in public health pre-dating the founding of our college in 1999. The College of Public Health launched two undergraduate degree programs in public health in 2016. The Bachelor of Arts and Bachelor of Science degrees in public health provide students with a basic understanding of the five core public health knowledge areas: biostatistics, social and behavioral sciences, epidemiology, health policy and management, and occupational and environmental health sciences. The program was re-accredited by CEPH in 2018.

The inaugural class of public health majors included 32 students admitted directly from high school. In 2017, we began admitting current University of Iowa students into the major. There are currently 162 undergraduate public health majors at the University of Iowa, including 50 new first-year students admitted directly from high school, 72 second-year students, and 40 third-year students. Our student population is evenly divided between the Bachelor of Arts and Bachelor of Science degree programs. We will be graduating our first cohort in Spring 2020. Our enrollment goal is approximately 500 undergraduate students in total, with 125 students per graduating cohort.

### Supporting Personnel

The Undergraduate Program Office at the University of Iowa College of Public Health is staffed by a faculty program director (MC), a full-time professional staff academic advisor (KK), and a full-time professional staff recruiter/admissions specialist. Curricular decisions for the undergraduate program are supported by the Undergraduate Program Committee which represents faculty from each department, staff, and both graduate and undergraduate students.

## Learning Objectives

The experiential learning program was developed with the following programmatic objectives in mind, which map back to CEPH foundational competencies, cross-cutting concepts, and experiential activities for undergraduate public health students. Students should be able to:

- thoughtfully consume, synthesize, and evaluate scientific information pertaining to public health
- communicate and translate public health information and science through a variety of media to a broad and diverse audience
- gain practical experience in public health practice and/or public health research
- distinguish the cultural contexts in which public health professionals work

## Pedagogical Format

### Overview and Expectations

Our public health majors attain experiences in public health research and/or practice by completing the experiential learning degree requirement. Students must complete at least one of the following experiential learning activities before graduation: public health research, public health internship, service learning courses related to public health, or global learning. These activities are situated outside of the course-based curriculum; however, we have assigned course numbers and titles to each experience so we are able to track student participation and completion.

It is highly recommended that students first complete CPH:2050 Second Year Undergraduate Public Health Seminar which provides an overview of experiential learning expectations and procedures as well as professional development opportunities that will help students to identify, apply for, and complete experiential learning activities. Students are encouraged to complete more than one experiential learning activity while in the degree program, when possible. Students are responsible for finding their own experiences, with support by the Undergraduate Program Office. Students must be in good academic standing during the semester in which they complete an experiential learning activity.

All students, regardless of the chosen experience, must: (1) Submit an application in order to request permission to register for academic credit, prior to the start of their experience; (2) Complete an early-term self-assessment and also be evaluated by their supervisor; (3) Submit a final written report; (4) Present their experiences in an oral or poster format; and (5) Complete an end-of-term self-assessment and also be evaluated by their supervisor. At the end of the experience, students receive of grade of “satisfactory” or “fail.” Final grade assignment is made by the Undergraduate Program Director. An overview of our experiential learning program can be found online^8^.

#### Application and Registration

At the time of application for credit and prior to the start of the experience, students are expected to develop a personal learning plan which identifies (1) what the student will learn during the course of the experience, (2) how these learning objectives integrate with their personal educational and career goals, and (3) what specific actions, processes, and work assignments will allow the student to achieve each objective. This personal learning plan is integrated into our online application form. The application form is signed by the student's supervisor and returned to the Undergraduate Program Office for review. Student applications are reviewed and approved by the undergraduate public health academic advisor and the undergraduate program director. Once approved, the student may register their experiential learning activity. Students may register for variable credit (0–3 semester hours), however, a minimum of 80 h of activity is required for all experiences. Once registered, students gain access to an online course site in our learning management system, which we use to communicate with students regarding further assignment instructions and deadlines.

#### Student Evaluation–Skills and Professionalism

Approximately 2–4 weeks into the start of each experience, we communicate with students and their supervisors for a quick check-in via an electronic survey. The student evaluation provides an opportunity for students to evaluate their experience to-date and serves as an early check-in to address any concerns thus far about their experience. The evaluation includes questions about satisfaction with the experience and the supervisor, consistency of expectations with respect to duties as outlined in their application, and identification of unexpected challenges. Likewise, we ask the student's supervisor if the student is showing up and meeting expectations, is experiencing any significant challenges (including those related to performance and conduct), and is accepting and following guidance and supervision. We also encourage the supervisor to provide early feedback to the student. If concerns are identified on either evaluation, the Undergraduate Program Office will work with the student and supervisor to identify a plan to address these concerns.

At the conclusion of the experience, we once again communicate with students and their supervisors for a final evaluation of performance. The students are asked to evaluate their work, performance, and attitude which includes items related to learning, communication skills, problem solving skills, professional development skills, interpersonal and teamwork skills, organizational effectiveness, basic work habits, and professional conduct. This activity provides the student an opportunity to reflect on their experience holistically and to have the Undergraduate Program Office analyze their performance, learning, and professional development, which is used in assessing a grade. This evaluation also includes questions for the student to evaluate their supervisor. We encourage students to share their feedback and observations with their supervisor. Student supervisors are asked to evaluate students on the same items on work, performance, and attitude as were included on the student self-evaluation. Additionally, we ask open-ended questions about student strengths and weaknesses. We encourage supervisors to share their feedback and observations with the student.

#### Student Evaluation–Final Student Products

At the end of the experience, we require that students submit a 4–5 page written report. This report includes the following sections: (1) Introduction—a background of the experience including a description of the public health issues being addressed and their significance, and how the student's experience fits with the overall research purpose (if engaged in research) or the mission of the organization (if engaged in an internship or service learning course); (2) Project Description—including but not limited to project goals, activities, student role, unanticipated challenges, and significant accomplishments; (3) Personal Assessment—including how the experience has contributed to personal and professional goals (including preparation through completed coursework and skill development); understanding of public health, and attainment of experiential learning activity learning objectives; and (4) Conclusion and Future Impact—a description of the new knowledge gained through the experience and how it may impact the student in the future, including how this experience complements the student's remaining studies, campus involvement, and post-baccalaureate plans.

Students are also required to present their activities through either an oral or poster presentation in a venue pre-approved by their supervisor and/or the Undergraduate Program Office. There are numerous opportunities for students to present at the College of Public Health, the University of Iowa, statewide, or nationally. Additionally, each semester the College of Public Health Undergraduate Program Office hosts an experiential learning fair. We encourage all students who have completed an experience to present at this fair, which is attended by current undergraduate and graduate students, faculty, staff, and community practice partners.

### Overview of Experiential Learning Activities

#### Public Health Research

Engaging in public health research allows students to enrich their educational experience by integrating coursework with real-life experiences, establishing personal mentored relationships with faculty members, and applying their knowledge to make research contributions that positively impact communities. Students have two options to fulfill this experiential learning opportunity and may conduct their research at the College of Public Health, the University of Iowa, or elsewhere. CPH:3999 Undergraduate Research Experience in Public Health allows hands-on undergraduate involvement in ongoing scholarly public health research activities under the supervision of faculty, research staff, postdoctoral scholars and fellows, or graduate students (with supervision by a faculty member). Independent student research projects are not an expectation for this activity. CPH:4990 Mentored Independent Undergraduate Research in Public Health provides the framework for an independent student research project under the supervision of a faculty mentor. Undergraduate public health majors who plan to graduate with honors in the major are required to register for CPH:4990 in order satisfy the honors thesis requirement.

#### Public Health Internship

Through public health internships, students have the chance to apply classroom lessons to real-world public health issues as they work with professionals, organizations, and populations. Internships provide an opportunity to explore future careers, develop transferrable skills, establish a professional network, and gain valuable experiences and accomplishments to add to their resume. Students may complete internships locally, nationally, and globally in both traditional and non-traditional public health settings. Students register their internships under CPH:4850 Undergraduate Public Health Internship.

#### Service Learning in Public Health

Service-learning courses allow students to integrate, synthesize, and apply classroom learning by combining rigorous academic coursework with community engagement. Students register their service learning courses under CPH:3750 Undergraduate Service Learning in Public Health. To date, we have only accepted service learning courses already established at the University of Iowa which includes local, national, and global opportunities.

#### Global Learning in Public Health

Students are encouraged to pursue opportunities to work with globally diverse populations in a global (i.e., study abroad) or local context (i.e., study away). Global learning encourages students to explore cultures, life experiences, and perspectives that are different from their own. Students may pursue global research, global internships, pre-approved study abroad programs provided by third-party vendors that combine public health coursework with either internships, service learning, or independent study opportunities, or local/global opportunities with College of Public Health faculty. Completing public health coursework in an international setting (without additional experiential activities) does not meet the expectations of experiential learning. Students register these experiences under CPH:4750 Undergraduate Global Learning in Public Health.

## Experience to Date

To date, 22 (13.6%) of 162 public health majors have completed their experiential learning requirement. This includes 0 of 50 first-year students, 6 (8.3%) of 72 second-year students, and 16 (40%) of 40 third-year students. At least one student has completed each of the four experiential learning activity categories: 6 students have completed internships, 15 students have completed research experiences (all of which were registered as CPH:3999), 1 student has completed a service learning course, and 1 student has completed a global learning experience. More students complete research opportunities during the academic years (14 of 15 students, 93%), as compared to the summer term. While more students complete internships during the summer term (6 of 6 students, 100%), as compared to the academic year.

When surveying second-year students who have completed CPH:2050 Second Year Undergraduate Public Health Seminar, we find that students are very interested in internships and global learning opportunities and somewhat interested in research and service learning opportunities.

Students have completed internships with local, state, and national public health agencies, as well as non-profit organizations. Students have primarily conducted research with College of Public Health faculty members. Students interested in global learning opportunities have worked closely with the university's Study Abroad office and the college's Global Public Health Coordinator to find study abroad opportunities that include experiential learning components, including internships, research, and service learning.

Current evaluation of the experiential learning program is limited to student and supervisor evaluations. Future assessment plans include a survey of graduating students and recent graduates who are approximately 1 year out of the program to determine what impact the experiential learning program has had on their professional development, short-term educational and career plans, and their level of perceived preparation for and success in their current placement.

## Discussion

The goal of our experiential learning program is to foster the application of concepts and skills learned in the classroom to real-world public health situations in order to prepare students to join the public health workforce, further their education, or apply this knowledge to other areas of their future personal and professional lives within or outside of the public health field. The experiential learning program at the University of Iowa College of Public Health is separate from our cumulative capstone course. This was an intentional decision. With early experiential learning opportunities, students are able to (1) identify and try out areas of interest, (2) accumulate multiple experiences over time, (3) develop technical and transferrable skills that can be applied to their remaining coursework, and (4) speak from experience when applying for graduate and professional programs or interviewing for jobs. Additionally, this program supports CEPH foundational competencies and cross-cutting concepts in a way that is adaptable to student interests and perhaps more flexible than if were intended to be both culminating and experiential.

### Recommendations

Should other schools and programs be interested in implementing similar experiential learning programs, we offer the following recommendations.

- When possible, include a required, preparatory course in the curriculum. Our undergraduate office is small with respect to staff and other resources. In anticipation of growth in our undergraduate program, we incorporated a required seminar course in our curriculum to provide the necessary tools and skills so that our students may be confident in self-identifying opportunities that are of interest to them, applying and competing for those opportunities, and completing them successfully.
- Track students through the various stages of the process using formal course numbers and titles, databases, online application and evaluation forms, and a robust learning management system. We have found that moving away from paper-based record keeping and increasing efficiencies in our online application system have reduced administrative burden.
- Build in opportunities for flexibility and creativity. This is particularly important for undergraduate programs that are situated within schools or programs of public health which also require applied learning experiences for graduate students. This is also important for programs situated in geographical locations that are either limited in traditional public health opportunities or are competing with several other health-related majors or schools and program of public health. Experiences should be related to public health, but could occur in traditional or non-traditional public health settings. We encourage our collegiate faculty to supervise undergraduate research; however, we also encourage our students to look beyond our college for research mentors who are conducting impactful public health research.
- Work with campus partners. Many universities and colleges may have centralized offices to support students in internship/career placement, study abroad/away, and/or undergraduate research.

### Final Thoughts

The purpose of this article was to share one model for the creation of an experiential learning program for undergraduate public health students. Experiential learning is a high impact educational activity that supports student success and can be tailored to meet the needs of the student and the program. We encourage other schools and programs of public health to share their experiences.

## Author Contributions

MC conceived the initial concept, assisted in program design, development and implementation, drafted the manuscript, and provided critical review and revision of the manuscript. KK assisted in program design, development and implementation, and provided critical review and revision of the manuscript.

### Conflict of Interest Statement

The authors declare that the research was conducted in the absence of any commercial or financial relationships that could be construed as a potential conflict of interest.

## Footnotes

^1^https://storage.googleapis.com/media.ceph.org/wp_assets/2016.Criteria.pdf

^2^https://storage.googleapis.com/media.ceph.org/wp_assets/2018.SBPcriteria.pdf

^3^http://www.aspph.org/educate/framing-the-future/

^4^https://www.aacu.org/leap/hips

^5^https://www.aee.org/what-is-ee

^6^https://www.nsee.org/8-principles

^7^https://provost.uiowa.edu/sites/provost.uiowa.edu/files/wysiwyg_uploads/StrategicPlan_2021.pdf

^8^https://www.public-health.uiowa.edu/experiential-learning-undergraduate/
